# Expression of *aldo-keto reductase family 1 member C1 (AKR1C1) *gene in porcine ovary and uterine endometrium during the estrous cycle and pregnancy

**DOI:** 10.1186/1477-7827-9-139

**Published:** 2011-10-20

**Authors:** Kyeong-Seok Seo, Purevjargal Naidansuren, Sang-Hwan Kim, Seong-Jo Yun, Jong-Ju Park, Bo-Woong Sim, Cha-Won Park, Tseeleema Nanjidsuren, Myung-Hwa Kang, Heewon Seo, Hakhyun Ka, Nam-Hyung Kim, Sue-Yun Hwang, Jong-Taek Yoon, Keitaro Yamanouchi, Kwan-Sik Min

**Affiliations:** 1 Animal Biotechnology, Graduate School of Bio & Information Technology, Institute of Genetic Engineering, Hankyong National University, Ansung 456-749, Korea; 2 Department of Food and Nutrition, Hoseo University, Asan 336-795, Korea; 3 Division of Biological Science and Technology, Yonsei University, Wonju 220-710, Korea; 4 Department of Animal Science, Chungbuk National University, Cheongju 361-763, Korea; 5 Department of Veterinary Physiology, Veterinary Medical Science, The University of Tokyo, Tokyo 113-8657, Japan

## Abstract

**Background:**

The aldo-keto reductase family 1 member C1 (AKR1C1) belongs to a superfamily of NADPH-dependent reductases that convert a wide range of substrates, including carbohydrates, steroid hormones, and endogenous prostaglandins. The 20alpha-hydroxysteroid dehydrogenase (20alpha-HSD) is a member of AKR family. The aims of this study were to determine its expression in the ovary and uterus endometrium during the estrous cycle and pregnancy.

**Methods:**

Rapid amplification of cDNA ends (RACE) experiments were performed to obtain the 5' and 3' ends of the porcine *20alpha-HSD *cDNA. Reverse-transcriptase-PCR (RT-PCR), real-time PCR, northern blot analysis, and western blot analysis were performed to examine the expression of porcine 20alpha-HSD. Immunohistochemical analysis was also performed to determine the localization in the ovary.

**Results:**

The porcine 20alpha-HSD cDNA is 957 bp in length and encodes a protein of 319 amino acids. The cloned cDNA was virtually the same as the porcine *AKR1C1 *gene (337 amino acids) reported recently, and only differed in the C-terminal region (the *AKR1C1 *gene has a longer C-terminal region than our sequence). The *20alpha-HSD *gene (from now on referred to as *AKR1C1*) cloned in this paper encodes a deletion of 4 amino acids, compared with the C-terminal region of *AKR1C1 *genes from other animals. Porcine AKR1C1 mRNA was expressed on day 5, 10, 12, 15 of the cycle and 0-60 of pregnancy in the ovary. The mRNA was also specifically detected in the uterine endometrium on day 30 of pregnancy. Western blot analysis indicated that the pattern of AKR1C1 protein in the ovary during the estrous cycle and uterus during early pregnancy was similar to that of *AKR1C1 *mRNA expression. The recombinant protein produced in CHO cells was detected at approximately 37 kDa. Immunohistochemical analysis also revealed that pig AKR1C1 protein was localized in the large luteal cells in the early stages of the estrous cycle and before parturition.

**Conclusions:**

Our study demonstrated that AKR1C1 mRNA and protein are coordinately expressed in the luteal cell of ovary throughout the estrous cycle and in the uterus on day 30 of pregnancy. Thus, the porcine AKR1C1 gene might control important mechanisms during the estrous cycle.

## Background

The aldo-keto reductase (AKR) superfamily are monomeric oxidoreductases that catalyze the NADP(H)-dependent reduction of a wide variety of substrates, including steroids, prostaglandins, bile acids, carbohydrates, and xenobiotics [1]. A group of AKRs known as hydroxysteroid dehydrogenases (HSDs) play a pivotal role in the modulation and regulation of steroid hormones [2], such as androgens, estrogens, and progestins, and are thus considered important targets for drug design [3]. AKR1C1, a member of the AKR1C subfamily (that shows both 20α- and 3α-HSD activities), plays a major role in progesterone metabolism and maintenance of pregnancy through the formation of progestin. AKR1C1 also has high 20α-HSD activity [4]. Animal AKRs are involved in a wide range of cellular processes that include the biosynthesis of steroid hormones in classical steroidogenic tissues [1].

The products of AKR activity have been implicated in prostate disease, breast cancer, obesity, polycystic ovary disease, and delay in onset of puberty in humans [5,6]. *AKR1C *genotypes are associated with nipple number as well as having possible effects on age at puberty and ovulation rate in pigs [7]. In swine, the rate of pubertal development and successful pregnancy in gilts affects the efficient management of breeding females. Human AKR1C1 (20α-HSD) has been functionally expressed in the fission yeast, and that this has enabled the resulting yeast strain to efficiently catalyze the reduction of progesterone to 20α-dihydroprogesterone. Thus, the process of AKR-dependent whole-cell biotransformation has been established, to be used for the production of human AKR metabolites on a large scale [8].

A tissue distribution study has demonstrated that 20α-HSD is expressed in the placenta, but not in the adrenal gland, liver, or spleen during pregnancy. The *20α-HSD *mRNA is expressed in the uterus and fetal skin during pregnancy and has been suggested to play a role in maintaining pregnancy in goats [9]. The 20α-HSD enzyme plays a critical role in the regulation of luteal function and is also localized in the placenta of rats [10], goats [9], and humans [4]. Histochemical data have illustrated strong 20α-HSD activities in several large luteal cells but not in granulosa cells [11,12]. In ruminant animals, goat *20α-HSD *mRNA is mainly localized in the endometrial epithelium on the caruncle side of the placenta [13]. In pigs, all *AKR1C *genes are expressed in adult tissues (spleen, lung, ovary, adrenal, gland, kidney, and endometrium). Pig *AKR1C4 *is expressed in all tissues, and *AKR1C2 *is the only other *AKR1C *gene expressed in the brain [7]. The *AKR1C1 *gene is widely expressed in adult tissues, but is not expressed in the pancreas, pituitary gland, small intestine, or brain. However, the regulation of the temporal expression pattern of *AKR1C1 *in reproductive tissues (ovary and uterus) during the estrous cycle and pregnancy are not completely understood.

To gain further insights into the expression and localization of AKR1C1 in the porcine ovary and uterine endometrium during the estrous cycle and pregnancy, we analyzed the mRNA and protein using RT-PCR, real-time PCR, northern blot analysis, western blot analysis, and immunohistochemistry.

## Methods

### Materials

The cloning vector pCR2.1, expression vectors pcDNA3 and pcDNA4/HisMax, GeneRacer kit, and Super Script™ First-Strand synthesis system were purchased from Invitrogen Life Technologies (Carlsbad, CA). The GLASSMILK gel extraction kit was purchased from Q-BIO gene (Cambridge, UK). Restriction enzymes and DNA ligation kits were bought from Takara (Tokyo, Japan). The One-Step SYBR RT-PCR kit was purchased from Toyobo (Osaka, Japan). Pro-PREP™ protein extraction solution was purchased from Intron Biotechnology (Seoul, Korea). The Digoxigenin (DIG) DNA labeling kit and Lumi-Light western blot kit were bought from Roche (Roche, Germany). Horse serum, goat serum, the avidin-biotin-horseradish peroxidase (ABC) detection kit, 3,3'-diaminobenzidine (DAB), hematoxylin, and methyl green were purchased from Vector Laboratories (Burlinggame, CA). Anti-rabbit immunoglobulins and anti-rabbit Alexa 488 secondary antibody were bought from Dako Cytomation. *E-scherichia. coli *competent cells (ECOS) were obtained from Yeastern Biotech Co. Ltd. (Seoul, Korea). CHO-K1 cells were obtained from the Japanese Cancer Research Resources Bank (Tokyo, Japan). Ham's F-12, Opti-Minimal Essential Medium (MEM) I, serum-free CHO-S-SFM II, and Lipofectamine™ 2000 reagents were bought from Gibco BRL (MD, USA). Fetal bovine serum was from Hyclone Laboratories (UT, USA). The oligonucleotides were synthesized by Genotech (Daejon, Korea), and all other chemicals were obtained from local suppliers.

### Tissues

Ovary tissues were obtained by laparotomy of pigs under general anesthesia on days 0, 2, 5, 10, and 15 of the estrous cycle. Ovaries were also collected from pigs on days 0, 30, and 60 of pregnancy, and before parturition. Uteri were obtained from pigs on days 12 and 15 of the estrous cycle, and at days 12, 15, and 30 of pregnancy. Tissues were completely washed 2 times in ice-cold phosphate-buffered saline (pH 7.2) and dissected into small fragments. Tissues were snap-frozen in liquid nitrogen and stored at -80°C. The experiments were conducted according to the Guidelines for the Care and Use of Animals, Hankyong National University.

### Preparation of porcine AKR1C1 fragments

PCR primers were designed according to the previously reported homologous part of the *20α-HSD *cDNA sequence [4,9,13]. The specific primers (sense: 5'-GTG AAG AGA GAA GAC ATA TTC-3' and antisense: 5'-CCA CGT TGT ATC TGG TAG CGA AGG-3') were synthesized from the nucleotide sequence of the cloned *20α-HSD *gene. Total RNA was extracted using the Trizol reagent. cDNA synthesis was performed using the SuperScript™ First-Strand Synthesis System according to the manufacturer's instructions. PCR was performed with 2.5 units of pfu polymerase in Quick Thermo-II using the following parameters: 30 cycles of denaturation (91°C for 1 min), annealing (37°C for 1 min), and extension (72°C for 2 min). The PCR products were analyzed by electrophoresis in Tris-acetate containing EDTA (TAE). The PCR fragments (572 bp) were ligated into the pCR2.1 vector and sequence data were analyzed using computer software (DNASIS).

### 3'- and 5'-Rapid Amplification of cDNA Ends system

GeneRacer 3'-primer (5'-GCT GTC AAC GAT ACG CTA CGT AAC G-3'), and GeneRacer 3'-nested primer (5'-CGC TAC GTA ACG GCA TGA CAG TG-3') for 3'- rapid amplification of cDNA ends (RACE) were designed according to the nucleotide sequence of the resulting PCR product. The gene specific primer (GSP) GeneRacer 5'-primer (5'-CGA CTG GAG CAC GAG GAC ACT GA-3'), and the GeneRacer 5'-nested primer (5'-GGA CAC TGA CAT GGA CTG AAG GAG TA-3') for 5'-RACE were designed according to the nucleotide sequence of the resulting PCR product. The 3'- and 5'-RACE experiments were performed using the GeneRacer kit (Invitrogen, USA) and conducted according to the manufacturer's instructions. To confirm the sequence accuracy for the stop codon in the 3'-terminal region, we conducted the PCR with specific primers (sense: 5'-GTG AAG AGA GAA GAC ATA TTC-3' and antisense: 5'-GAA GTG TCT GCA CTT CTG AAA GCT-3').

### RT-PCR and real-time PCR of porcine *AKR1C1 *mRNA expression

RT-PCR was performed using the AccuPower RT-PCR kit (RT/PCR PreMix). Total RNA (1.0 μg) extracted from the pig ovary on days 0, 5, 10, and 15 of the estrous cycle was mixed with the reverse primer (5'-GCC ATT GCC AAA AAG CAC AAG-3'), incubated at 70°C for 5 min, and placed on ice. The forward primer (5'-GGA AAG CGG ATA GTC AGG GTG ATC-3') was then added, and the reaction volume was brought to 20 μL by adding diethylpyrocarbonate-distilled water (DEPC-DW). The cDNA synthesis reaction was performed using the following parameters: 42°C for 60 min and 94°C for 5 min. PCR was carried out according to the following parameters: 94°C for 1 min, followed by 30 cycles (94°C for 1 min, 56°C for 1.5 min, and 72°C for 1 min), followed by a final extension at 72°C for 8 min. Primers for glyceraldehyde-3-phosphate dehydrogenase (GAPDH) were used for the normalization of porcine *20α-HSD *expression and the primer sequences of the forward and reverse primers were 5'-ACC ACA GTC CAT GCC ATC AC-3' and 5'-TCC ACC ACC CTG TTG CTG TA-3', respectively. The expected length of the PCR fragment was 452 bp. The PCR conditions were 26 cycles for 10 s at 98°C, 20 s at 55°C, and 20 s at 72°C. Gel electrophoresis was used to analyze 10 μL of the PCR products. Real-time PCR was carried out using a One-Step SYBR RT-PCR kit. The real-time PCR amplification mixture consisted of the following reagents: a total of 5 μg of RNA, 1× of 2× One-Step SYBR RT-PCR buffer, 5 pmol of each primer, 2.5 U of Takara Ex *Taq *HS (Takara, Japan), 50 U of Moloney murine leukemia virus (M-MLV) RTase (RNase H free), and 20 U of RNase inhibitor. This mixture was added to PCR tubes, and the reaction volume was brought to 25 μL by adding RNase-free dH_2_O. The thermocycler profile was 10 min at 95°C; 30 cycles of 10 s at 95°C, 15 s at 56°C, and 20 s at 72°C; followed by 45 s at 72°C.

### Northern blotting analysis

For northern blot analysis, RNA electrophoresis was performed on an agarose gel containing 10' MOPS and 37% formaldehyde. The total RNA concentration from pig ovary and uterus was adjusted to 10 μg/μL. Following electrophoresis, RNA was transferred overnight to a membrane with 20' SSC. The probe was prepared by purifying the sample after PCR amplification. Probe labeling was performed with the DIG DNA Labeling Kit. The membrane was prehybridized for 1 h at 68°C and hybridized at 68°C overnight with DIG-labeled pig AKR1C1 cDNA with gentle rocking. The membrane was subsequently washed 2 times with buffer (2' SSC/0.1% SDS) at 68°C for 5 min with gentle rocking, and 2 times with 0.5' SSC/0.1% SDS at 68°C for 15 min with gentle rocking. The anti-DIG antibody (5 μL) was mixed with blocking reagent, added to the membrane, and incubated at room temperature (RT) for 1 h. The membrane was washed 2 times with washing buffer at RT for 15 min, and equilibrated with detection buffer at RT for 5 min. The band was detected by the addition of CDP-Star reagent.

### Western blot analysis

Total protein was extracted using the PRO-PREP™ protein extraction solution. About 10-20 mg of ovarian and uterus tissues was used. The samples were then homogenized in 600 μL of PRO-PREP™ solution, and cell lysis was induced by incubation on ice for 30 min. The tube was centrifuged at 13,000 rpm at 4°C for 5 min, and the supernatant was transferred to a fresh 1.5-mL tube. Protein concentration was measured using the Bradford protein assay [14]. Samples were subjected to SDS-PAGE and transferred to a polyvinylidene fluoride (PVDF) membrane (0.2 μm) through a semidry electroblotter apparatus. The membrane was blocked with 1% blocking reagent for 1 h and incubated with a 1:1,500 dilution of bovine-specific polyclonal 20α-HSD antibody for 1 h. The membrane was washed to remove unbound antibody and incubated with a 1:2,000 dilution of a secondary antibody linked to anti-rabbit IgG-peroxidase (POD) for 30 min. Next, the membrane was incubated for 5 min with Lumi-Light substrate solution (2 mL), covered with plastic wrap, and exposed to X-ray film for 1-10 min.

### Construction of expression transfer vector and transient transfection of CHO cell lines

The full-length pig *AKR1C1 *cDNA was PCR-amplified with specific primers containing a 5'-end *Xho*I restriction site in the *AKR1C1 *cDNA sequence, and a 3'-end *Xba*I site in the sequence cloned in this study. The PCR fragments were ligated into the PCR2.1 vector and sequenced. The fragments digested with *Xho*I and *Xba*I restriction enzymes were ligated into eukaryotic expression vectors (pcDNA3 and pcDNA4/His Max) that had been digested with *Xho*I and *Xba*I (designated as pcDNA3-pAKR1C1 and pcDNA4/His Max-pAKR1C1). Each vector was completely sequenced to confirm the presence of the Kozak site and to rule out the possibility of PCR errors. CHO-KI cell lines were cultured in growth medium (Ham's F12 medium containing penicillin [50 U/mL], streptomycin [50 mg/mL], glutamine [2 mM], and 10% fetal calf serum). The cells were incubated at 37°C in 5% CO_2_. Cultured CHO-K1 cells were transfected with the expression vectors by using the liposome transfection method described previously [15]. Briefly, DNA (0.8 μg) was diluted in 50 μL of Opti-MEM I reduced medium without serum and mixed gently. Lipofectamine 2000 (2 μL) was added gently to a separate 50-μL aliquot of Opti-MEM I medium. Each tube was incubated separately for 5 min at RT, after which the diluted DNA and the diluted Lipofectamine 2000 were combined into a single tube. The 2 samples were mixed gently and incubated for 20 min. A 100-μL aliquot of DNA-Lipofectamine 2000 complex was added to each well, and the samples were incubated for 4-6 h, and then, 250 μL of 20% FBS was added to each well. The cells were incubated at 37°C in a CO_2 _incubator for 24 h. The following day, transfected cells were washed 2 times, placed in 500 μL of serum-free medium, and incubated at 37°C for 48 h. Next, the culture medium was removed and the cells were collected into a tube. Subsequently, the cells were centrifuged at 15,000 rpm for 10 min, and the cell debris was recovered.

### Immunoblot analysis of recombinant pig AKR1C1 protein

Recombinant protein was extracted using PRO-PREP protein extraction solution. Recombinant AKR1C1 proteins were subjected to SDS-PAGE and transferred to a PVDF membrane through a semidry electroblotter apparatus. The western blot analysis method described above was followed.

### Immunohistochemistry

Immunohistochemical staining was performed using the Vectastain ABC kit. Ovary samples taken on days 2, 5, 10, and 15 of the estrous cycle and before parturition were fixed in 10% neutral buffered formalin at RT for 24 h and washed with PBS. Next, the fixed samples were dehydrated in graded ethanol (EtOH) (3 min each in 50%, 2'; 70%, 1'; 95%, 1(; 100%, 1() and dealcoholized with xylene for 2 h and embedded in paraffin. Paraffin-embedded tissues were sectioned at 8 μm and mounted onto poly l-lysine-coated slides and dried at RT. The slides were deparaffinized and rehydrated (3 min each in xylene, 2(; 100% EtOH, 2(; 95% EtOH, 1(; 70% EtOH, 1(; 50% EtOH 1() and kept in the cold tap water. The slides were boiled in 10 mM sodium citrate for 10 min, left on ice for 20 min, and then washed in 3% hydrogen peroxide for 10 min and blocked for 1 h at RT. The slides were first incubated with the primary antibody for overnight for overnight at 4 C, followed by the anti-rabbit secondary antibody for 2 h at RT. Finally, the slides were immunostained using the ABC detection kit, stained with DAB, and observed under a Nikon Eclipse TE-2000-E confocal microscope.

### Data and statistical analysis

One - Way ANOVA Newman-Keuls Multiple Comparison tests were used to compare results between control and samples, using GraphPad Prism 5 (GraphPad Software). Asterisks indicate significant differences from the control group. (*p < 0.05, **p < 0.01, ***p < 0.001).

## Results

### Determination of AKR1C1 C-terminal region nucleotide sequence

**Figure 1:**
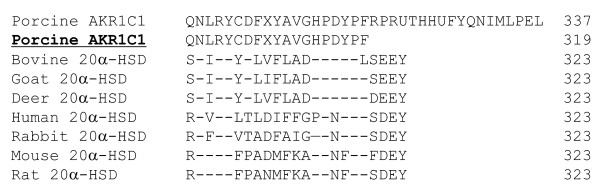
**Amino acid alignment of pig AKR1C1 and other animal 20α-HSD proteins**. A C-terminal amino acid region of pig AKR1C1 sequence was compared that of bovine, goat, deer, human, rabbit, mouse, and rat 20α-HSD. Dashed lines denote the amino acids identities. The numbers denote the amino acids before the stop codon. Porcine AKR1C1 denotes the results reported previously [7]. Bold and underlined AKR1C1 was shown sequence cloned in this paper.

The complete cDNA sequence (957 bp) for porcine *AKR1C1 *was obtained by assembly of the sequences from the PCR and RACE fragments. A BLAST search of the porcine reference genome sequence in the NCBI database by using the porcine *AKR1C1 *cDNA did not help identify the corresponding gene sequence. The porcine *AKR1C1 *cDNA encodes a protein of 319 amino acids (GenBank: JN62505). Based on the results of 3'-RACE, we detected a stop codon in a different site from that of porcine *AKR1C1 *reported previously [7]. We sequenced 20 clones from the 3'-RACE experiment to confirm the sequence accuracy. This result was different from the 337 amino acids sequence of pig AKR1C1 that was previously reported, and there was a deletion of 4 amino acids compared with other animal *20α-HSD *sequences reported to date (Figure 1).

### Expression of AKR1C1 mRNA by RT-PCR and real-time PCR

**Figure 2:**
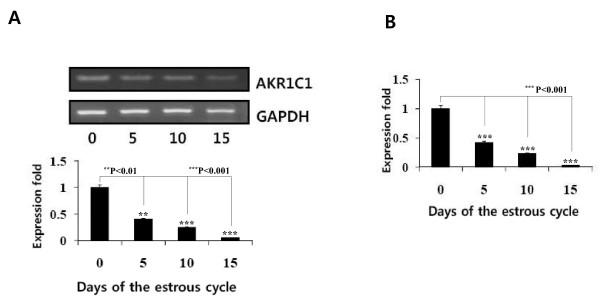
***AKR1C1 *mRNA expression in the ovary during the estrous cycle**. **A**. *AKR1C1 *mRNA was detected by RT-PCR. **B**. Real-time PCR results. Ovarian tissues were obtained by laparotomy under general anesthesia on days 0, 5, 10, and 15 of the estrous cycle. Total RNA was extracted and then subjected to RT-PCR and real-time PCR. The amplified products of the *AKR1C1 *and *GAPDH *genes were separated on agarose gel and stained with ethidium bromide. Representative results are shown; graphs show the average ± SEM of 3 independent experiments.

By using the specific primers for porcine *AKR1C1 *and amplification by RT-PCR and real-time PCR, porcine AKR1C1 mRNA was expressed on day 5, 10, 12, 15 of the cycle and 0-60 of pregnancy in the ovary (Figure 2A, B). The RT-PCR and real-time PCR results were almost the same. The *AKR1C1 *mRNA was detected in all ovarian tissues during the estrous cycle. However, the expression of *AKR1C1 *mRNA was particularly strong in the ovary on day 0 of the estrous cycle. mRNA expression in the ovary gradually decreased and was very low in the ovary on day 15 of the estrous cycle.

### Northern blot analysis

**Figure 3:**
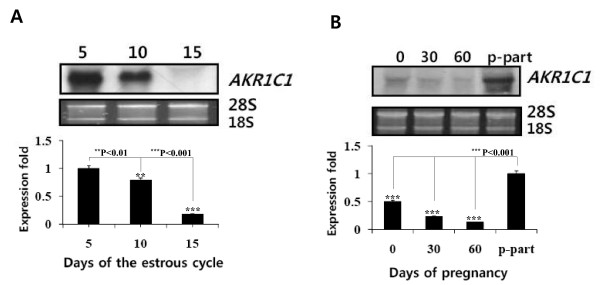
**Northern blot analysis of pig *AKR1C1 *mRNA expression in the ovary during the estrous cycle and pregnancy**. **A**. Ovarian tissues were obtained by laparotomy under general anesthesia on days 5, 10, and 15 of the estrous cycle. **B**. Ovaries were collected by the same method on days 0, 30, and 60 of pregnancy, and before parturition. Blots shown are the results of a representative experiment; graphs show the average ± SEM of 3 independent experiments. P-part: before parturition.

**Figure 4:**
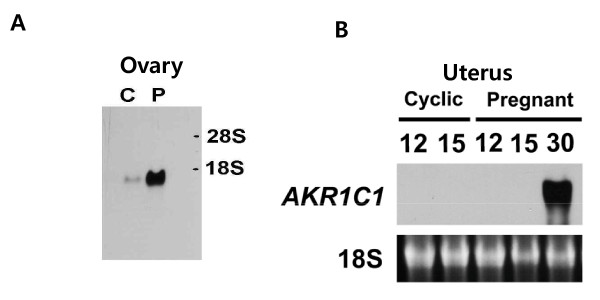
**Northern blot analysis of pig *AKR1C1 *mRNA expression in the ovary and uterus during the estrous cycle and pregnancy**. **A**. Total RNA was isolated from ovaries on day 12 of the estrous cycle and pregnancy. **B**. Uteri were collected on days 12 and 15 of the estrous cycle, and on days 12, 15, and 30 of pregnancy. Blots shown are the results of a representative experiment; graphs show the average ± SEM of 3 independent experiments. C: estrous cycle; P: pregnancy.

For northern blot analysis, total RNA was extracted from porcine ovarian tissues on days 5, 10, and 15 of the estrous cycle, and on days 0, 30, and 60 of pregnancy and before parturition. Northern blot analysis revealed a 1.2-kb mRNA in the ovary on day 5 of the estrous cycle and before parturition (Figure 3A). This pattern was very similar to the RT-PCR and real-time PCR results. This mRNA was more intensively expressed in the pre-parturition ovary than in the ovary at any other period during pregnancy (Figure 3B). Next, we analyzed the expression difference in the ovary on day 12 of the estrous cycle and pregnancy. An intense pig *AKR1C1 *mRNA band was detected with a size of approximately 1.2 kb in the ovary on day 12 of pregnancy (Figure 4A). Moreover, we also specifically detected *AKR1C1 *mRNA in the uterus on day 30 of pregnancy, but it was not detected in the uterus on days 12 and 15 of the estrous cycle and on days 12 and 15 of pregnancy (Figure 4B).

### Western blot analysis of pig ovary tissues

**Figure 5:**
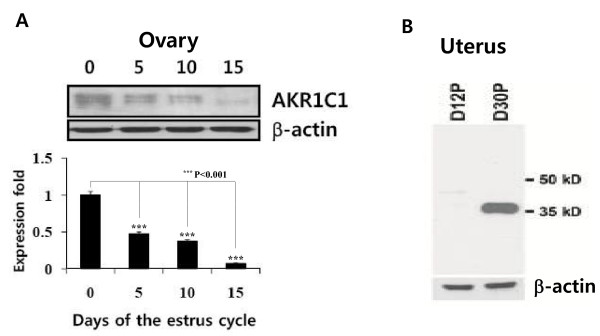
**Western blot analysis of pig AKR1C1 protein in the ovary during the estrous cycle and pregnancy**. **A**. Pig ovaries on days 0, 5, 10, and 15 of the estrous cycle were obtained by laparotomy under general anesthesia. **B**. Uteri were collected on days 12, and 30 of pregnancy. The proteins were transferred onto a PVDF membrane. Proteins on the blot were detected with rabbit anti-bovine 20α-HSD antibody, followed by staining with secondary antibody linked to anti-rabbit IgG-peroxide. M, marker; p-part, before parturition.

Pig AKR1C1 protein was detected at approximately 37 kDa by western blot analysis, using a specific anti-bovine 20α-HSD antibody developed in our laboratory. AKR1C1 protein was detected in all ovaries during the estrous cycle. The highest expression of the protein was detected on day 0 of the estrous cycle. Thereafter, its expression gradually decreased. The level of the protein was remarkably decreased in the ovary on day 15 of the estrous cycle (Figure 5A). Comparison of the level of the AKR1C1 protein with that of the mRNA in ovaries during the estrous cycle showed a similar pattern. Interestingly, 2 protein bands were detected at approximately 37 and 39 kDa in the ovarian tissues during estrous cycle (Figure 5A). Next, we analyzed the expression in the uterus on days 12 and 30 of pregnancy. An intense protein band was detected with a size of approximately 37 kDa in the uterus on day 30 of pregnancy (Figure 5B). But, it was not detected in the uterus on day 12 of pregnancy.

### Expression of recombinant AKR1C1 in the CHO-K1 cell line

**Figure 6:**
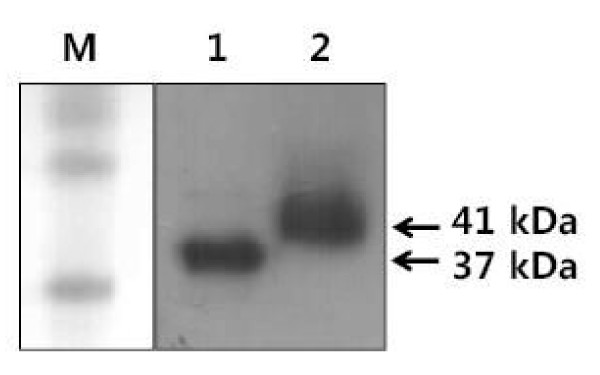
**Immunoblot analysis of AKR1C1 recombinant protein in CHO-K1 cells**. The bands corresponding to the protein produced by pcDNA3 + pAKR1C1 and pcDNA4/HisMax + pAKR1C1 vectors were detected. After gel electrophoresis, the proteins were transferred to a nitrocellulose membrane. The protein on the blot was detected with rabbit anti-bovine 20α-HSD, followed by staining with anti-rabbit IgG-POD. Lane 1, pcDNA3 + pAKR1C1; lane 2, pcDNA4/HisMax + pAKR1C1.

Two expressing vectors (pcDNA3 + pig *AKR1C1 *and pcDNA4/HisMax + pig *AKR1C1*) were transiently transfected into CHO-K1 cells. The cell lysates were collected and subjected to SDS-PAGE. Bovine 20α-HSD-specific antibody was used to detect the recombinant protein. The recombinant protein produced by the pcDNA4/HisMax + pig *AKR1C1 *expression vector has a tagging protein of 4 kDa. Thus, the protein produced in this vector was detected at about 41 kDa. The recombinant protein of 37 kDa was produced by the pcDNA3 vector (Figure 6).

### Immunohistochemical localization of AKR1C1 protein in the ovary during the estrous cycle and before parturition

**Figure 7:**
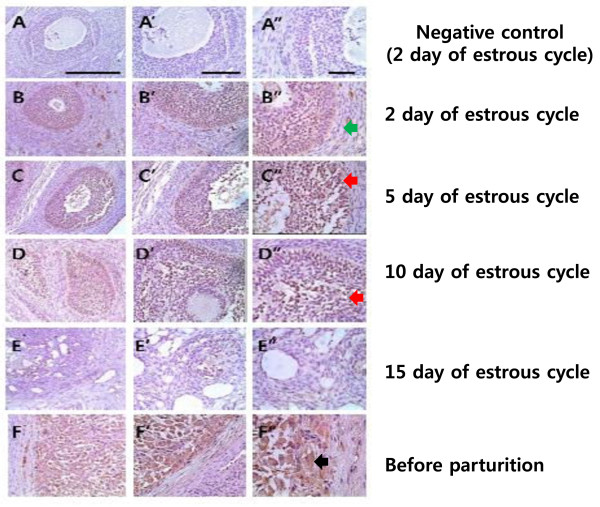
**Immunohistochemical localization of pig AKR1C1 protein in the ovary on days 2, 5, 10, and 15 of the estrous cycle and before parturition**. **A**. Ovary on day 2 of the estrous cycle (negative control). **B**. Ovary on day 2 of the estrous cycle. **C**. Ovary on day 5 of the estrous cycle. **D**. Ovary on day 10 of the estrous cycle. **E**. Ovary on day 15 of the estrous cycle. **F**. Ovary before parturition. Representative immunohistochemical analyses are shown for anti-bovine 20α-HSD (1:1,000). Anti-rabbit IgG (1:500) was used as a secondary antibody. For the negative control, normal rabbit serum (1:100) was used instead of the secondary antibody. Scale bar: 200 um (A, B, C, D, E, and F); 100 um (A', B', C', D', E', and F'), and 50 um (A'', B'', C'', D'', E'', and F''). Green arrow indicated the granulose layer and black arrow indicate the large luteal cells. Red arrow indicated the luteal cells.

To determine the cell types responsible for AKR1C1 protein expression in the ovary, we performed immunohistochemical analysis in the ovary on days 2, 5, 10, and 15 of the estrous cycle and before parturition. As shown in Figure 7, the AKR1C1 protein was localized in large luteal cells. It was intensely expressed in the luteal cells on days 2 and 5 of the early estrous cycle (Figure 7B, C). It was also strongly localized in the luteal cells of ovary at before parturition (Figure 7F). However, its signal was not detected in the small luteal cells.

## Discussion

In the present study, we determined the expression and localization of porcine AKR1C1 in the ovary and uterine endometrium through RT-PCR, real-time PCR, northern blotting, and immunohistochemistry during the estrous cycle and pregnancy. Analysis of the nucleotide sequence by using the GenBank database revealed that porcine *AKR1C1 *cDNA belongs to the AKR family. Both nucleotide and amino acid sequences of the porcine AKR1C1 cloned in this study showed high homology with those of bovine (86/82%), goat (80/78%), rat (76/66), mouse (76/68%), and human (81/76%) 20α-HSD. Based on the results of 3'-RACE, we detected a stop codon in a different site from that of porcine *AKR1C1 *reported previously [7]. Several conserved sequence patterns were found in the porcine *AKR1C1 *cloned in the present study. A catalytic tetrad, such as that consisting of Asp 50, Tyr 55, Lys 84, and His 117, is a common feature of the AKR family [1]. Other amino acids such as Gly 22, Gly 45, Asp 112, Pro 119, Gly 164, Asn 167, Pro 186, Gln 190, and Ser 271 are strictly conserved in the primary structure of all members of the AKR family [2]. Thus, these amino acids may play a role in conferring the appropriate tertiary structure necessary for the functional activity.

The present study confirmed that porcine *AKR1C1 *mRNA is expressed in ovarian tissues during the estrous cycle. This pattern was similar to the results of RT-PCR and real-time PCR results. As reported recently, porcine *AKR1C1 *gene is widely expressed in adult tissues as determined in a PCR study by Nonneman *et al*. [7]. However, these authors did not report on the expression level in the ovary during the estrous cycle and pregnancy. Although porcine *AKR1C1 *mRNA is expressed in several tissues, our findings suggest that the expression level of *AKR1C1 *mRNA in the ovary is different in the estrous cycle and in pregnancy. *AKR1C *genotypes are associated with nipple number [16], as well as possibly having effects on age at puberty and ovulation rate in pigs [7,17].

The expression of pig *AKR1C1 *mRNA was very different from that of bovine and goat 20*α*-HSD during the estrous cycle. The bovine 20*a*-HSD level was remarkably higher in the corpus luteum during the late estrous cycle (unpublished results). On the other hand, goat 20α-HSD expression was found in the corpus luteum during the late estrous cycle and expressed in the placenta and intercaruncular region of the uterus during mid to late pregnancy, but not in the adrenal gland, liver, or spleen [9,13]. However, human *AKR1C1 *(20α-HSD) mRNA is highly expressed in the liver, mammary gland, and brain, and is expressed at a low levels in the prostate, testis, adrenal gland, and uterus [4]. Albarracin *et al*. [18] indicated that the pattern of 20α-HSD mRNA expression in the corpus luteum closely paralleled the ontogeny of 20α-HSD enzyme activity. Pig *AKR1C1 *mRNA was also strongly expressed in the ovaries on day 12 of pregnancy and in the uterine endometrium on day 30 of pregnancy. Moreover, these results may provide insight into the role of the *AKR1C1 *gene at the abovementioned phases of the estrous cycle and pregnancy. However, the functions of AKR1C1 in the ovary and uterus during the early estrous cycle and pregnancy in pigs are not known in detail. In the present study, we suggest that porcine AKR1C1 plays a pivotal role on oocyte ovulation and implantation in ovary and pregnancy maintenance of fetus in uterus.

On the other hand, *20α-HSD *mRNA in goats was mainly localized in the endometrial epithelium on the caruncle side of the placenta on day 130 of pregnancy [13]. In mice, *in situ *hybridization analysis revealed that *20α-HSD *mRNA was localized in the endometrial epithelial cells, maternal placental endothelial cells, and fetal epidermal cells during pregnancy [19]. These findings may be related to differences in the distribution of 20α-HSD between these species. Steroid metabolites of AKR1C enzymes rise at the onset of puberty [20,21]. FSH secretion stimulates the development of antral follicles, and FSH levels are greater in some lines of gilts with higher ovulation rates [22,23]. Considerable support for an association between *AKR1C *genotypes and nipple number was detected, as well as some indication of an effect on age at puberty and possible ovulation rate [7]. Age at onset of puberty and ovulation rate at a specific age are negatively correlated traits in Meishan pigs because ovulation rate increases from puberty to later estrous cycles [24].

Pig AKR1C1 protein was most highly expressed in the ovary on day 0 of the estrous cycle, and was almost consistent with the mRNA expression pattern. This study shows that AKR1C1 protein and mRNA levels are expressed in a coordinated fashion in the ovary during the estrous cycle and pregnancy. However, this is not consistent with results in other animals, which show the presence of 20α-HSD in the ovary and placenta [9,11,12,25]. In bovines, its expression increased according to the phase of the estrous cycle (unpublished data). However, the pattern in pigs is in contrast with the expression of bovine and goat 20α-HSD. The isolated *AKR1C1 *cDNA was expressed in mammalian cells, and the protein product was detected by western blot at 37 kDa, identical to previous reports in other species: the baculovirus insect cell system in rat [25] and the *E. coli *system in goat, monkey, and rat [9,19,26]. Immunohistochemistry demonstrated that AKR1C1 is localized in large luteal cells. It was intensely expressed in luteal cells on day 5 of the early estrous cycle and before parturition. In cattle and deer, 20α-HSD was primarily expressed in large luteal cells of the corpus luteum during the late estrous cycle and pregnancy (unpublished data).

However, in pigs, the functions of AKR1C1 on ovulation have not been reported. This study represents the first report of specific expression levels and localization in the ovary and uterus during the estrous cycle and pregnancy period. Thus, further research is required to elucidate the function of AKR1C1 in the ovary and uterine endometrium during the estrous cycle and pregnancy.

## Conclusions

We identified the nucleotide sequence and expression pattern of the porcine AKR1C1. Our study demonstrated that AKR1C1 mRNA and protein are coordinately expressed in the ovary throughout the estrous cycle. Porcine AKR1C1 mRNA and protein also were highly expressed in the uterus on day 30 of pregnancy. AKR1C1 was primarily localized in large luteal cells during the early stages of the estrous cycle and in the ovary before parturition. Thus, the porcine AKR1C1 gene might control important mechanisms during the estrous cycle. Further studies are needed to determine the functional significance of porcine AKR1C1 during estrous cycle and pregnancy.

## Competing interests

The authors declare that they have no competing interests.

## Authors' contributions

KSS, PN, SHK, SJY, JJP, and BWS performed the experiments. CWP, TN, and HS performed IHC. MHK drafted the manuscript. HHK, NHK, SYH, JTY, and KSM designed the study, supervised the experimental work, and revised the manuscript. All authors read and approved the final manuscript. These authors contributed equally to this paper.
